# Identification of Target Genes Involved in Wound Healing Angiogenesis of Endothelial Cells with the Treatment of a Chinese 2-Herb Formula

**DOI:** 10.1371/journal.pone.0139342

**Published:** 2015-10-02

**Authors:** Jacqueline Chor Wing Tam, Chun Hay Ko, Chi Man Koon, Zhang Cheng, Wong Hing Lok, Ching Po Lau, Ping Chung Leung, Kwok Pui Fung, Wai Yee Chan, Clara Bik San Lau

**Affiliations:** 1 Institute of Chinese Medicine, The Chinese University of Hong Kong, Shatin, New Territories, Hong Kong; 2 State Key Laboratory of Phytochemistry and Plant Resources in West China, The Chinese University of Hong Kong, Shatin, New Territories, Hong Kong; 3 School of Biomedical Sciences, The Chinese University of Hong Kong, Shatin, New Territories, Hong Kong; University of Colorado Denver, UNITED STATES

## Abstract

Angiogenesis is vitally important in diabetic wound healing. We had previously demonstrated that a Chinese 2-herb formula (NF3) significantly stimulated angiogenesis of HUVEC in wound healing. However, the molecular mechanism has not yet been elucidated. In line with this, global expression profiling of NF3-treated HUVEC was performed so as to assess the regulatory role of NF3 involved in the underlying signaling pathways in wound healing angiogenesis. The microarray results illustrated that different panels of differentially expressed genes were strictly governed in NF3-treated HUVEC in a time-regulated manner. The microarray analysis followed by qRT-PCR and western blotting verification of NF3-treated HUVEC at 6 h revealed the involvement of various genes in diverse biological process, e.g., MAP3K14 in anti-inflammation; SLC5A8 in anti-tumorogenesis; DNAJB7 in protein translation; BIRC5, EPCAM, INSL4, MMP8 and NPR3 in cell proliferation; CXCR7, EPCAM, HAND1 and MMP8 in migration; CXCR7, EPCAM and MMP8 in tubular formation; and BIRC5, CXCR7, EPCAM, HAND1, MMP8 and UBD in angiogenesis. After 16 h incubation of NF3, other sets of genes were shown with differential expression in HUVEC, e.g., IL1RAPL2 and NR1H4 in anti-inflammation; miR28 in anti-tumorogenesis; GRIN1 and LCN1 in anti-oxidation; EPB41 in intracellular signal transduction; PRL and TFAP2A in cell proliferation; miR28, PRL and SCG2 in cell migration; PRL in tubular formation; and miR28, NR1H4 and PRL in angiogenesis. This study provided concrete scientific evidence in support of the regulatory role of NF3 on endothelial cells involved in wound healing angiogenesis.

## Introduction

Angiogenesis is vitally important in wound healing, which describes the formation of new blood vessels from the existing vasculature for restoring blood flow to wound tissues after injury for diffusion exchange of nutrients and metabolites. The luminal surface of circulation system in closely contact with blood is a single layer of endothelial cells (ECs) which are actively involved in the overall process of angiogenesis. It is tightly governed by pro- and anti-angiogenic factors and involves a cascade of signaling events [1]. Angiogenesis begins at the moment of injury. Resident ECs are responsive to a number of angiogenic growth factors for ECs activation after receptor binding. Four fundamental and essential steps of angiogenesis take place: (i) protease synthesis by ECs for degradation of basal lamina in the parent vessel associated with dissolving the tissue extracellular matrix (ECM); (ii) migration of ECs into the wound bed; (iii) proliferation and sprouting of ECs for restoring the tubular channels and forming the vascular loops; and finally (iv) remodeling and differentiation [2]. Normal wound healing is a highly orchestrated and dynamic mechanism which is comprised of the coordination of various cells including platelets, inflammatory cell, ECs, epidermal cells and parenchyma cells. The process involves hemostasis, coagulation, inflammation, regeneration, migration and proliferation of connective tissue associated with extracellular matrix proteins synthesis and deposition for enhancement of wound tensile strength. However, numerous studies reported that angiogenesis was seriously hampered in diabetes with diminished level of growth factors, persistent inflammatory response and ECs dysfunction [3]. Defects in the angiogenesis adversely affected the subsequent tissue granulation and healing process, leading to wound healing failure.

Astragali Radix (AR) and Rehmanniae Radix (RR) have long been used in Traditional Chinese Medicines (TCM) formulae and serve as the principal herbs in treating diabetic foot ulcer. Both clinical and scientific studies provided strong and equivocal evidence of AR and RR as alternatives to conventional medicines in the treatment of diabetic ulcer with wound modulating and pro-angiogenic effects. In the clinic, Wong *et al*. (2001) applied two herbal formulae F1 and F2 in promoting wound closure and preventing limb amputation in diabetic foot ulcer patients [4]. In the laboratory, Tam *et al*. (2011) demonstrated a modified herbal formula NF3 (AR and RR in the ratio of 2 to 1) that imposed promising wound healing effect in streptozotocin-induced diabetic foot ulcer rats [5]. Subsequent evidence further supported the pro-angiogenic capacity of NF3 in diabetic wound healing [6–9].

Previously, we had demonstrated that NF3 could significantly enhance the *in vitro* cell migration and tube formation ability of human umbilical vein endothelial cells (HUVEC) [5]. This revealed the pro-angiogenic capacity of HUVEC after NF3 treatment, promoting blood vessel formation in wound healing mechanism. However, the underlying mechanisms of the functional changes and modulation of the pathways had not yet been fully addressed. The advent of powerful technology of functional genomics has accelerated the investigations of gene identification and cellular function in a high-throughput and global scale. Making use of the advantages of DNA microarray technology, our ultimate aim was to identify the target genes involved in pathway resulting from the therapeutic effects of NF3.

In line with this, we focused on understanding the regulatory mechanisms of NF3 on HUVEC at transcriptional level. The 6 h and 16 h incubation of NF3-HUVEC chosen for microarray study were based on our previous study of pro-angiogenic activity of NF3-treated HUVEC on tube formation and migration scratch assay at 6 h and 16 h, respectively [5]. This study aimed to identify genes which were responsible for wound healing, as well as angiogenesis-associated pathways in different time frame. This would allow the identification of a large number of molecular targets closely related to the biological effects of NF3. Associated with the bioinformatic data mining tools, the identified target genes from microarray analysis of HUVEC would be further validated by qRT-PCR and western blotting so as to elucidate the role of NF3 in governing the angiogenic responses of the mature endothelial cells at the transcriptional level in wound healing angiogenesis.

## Materials and Methods

### Authentication and extracts preparation of NF3

Herbal formula NF3 was composed of Astragli Radix (AR) and Rehmanniae Radix (RR) in the ratio of 2:1 (w/w) and was prepared as described previously [5–7]. AR was derived from the dried root of *Astragalus membranaceus* (Fisch.) Bge., while RR was derived from the dried root of *Rehmannia glutinosa* Libosch. The raw herbs of AR and RR were purchased from mainland China. They were authenticated by morphological characterizations and thin layer chromatography in accordance with the Chinese Pharmacopoeia [10]. Voucher specimens of AR and RR were deposited in the museum of Institute of Chinese Medicine, The Chinese University of Hong Kong, with voucher specimen numbers: 2008–3201 for AR and 2008–3200 for RR.

For NF3 preparation, raw herbs of AR and RR were cut into small pieces and mixed in the ratio of 2:1 (w/w). After being soaked in 10 volumes of distilled water for 30 min, they were boiled under reflux for 1 h twice. The extracts were pooled, filtered and lyophilized into dry powder. The extraction yield was around 34% (w/w). The NF3 preparation was under strict quality control to ensure the identity throughout all the experiments. The chemical fingerprinting of NF3 was conducted by high performance liquid chromatography-mass spectrometry analysis and the details were described previously [11].

### Cell culture

The culture conditions of human umbilical vein endothelial cells (HUVEC) were followed by our previous method [5, 8]. In brief, HUVEC were subcultured to confluence in Dulbecco’s Modified Eagle Medium: Nutrient Mixture F-12 (DMEM/F12; Life technologies, USA) supplemented with 0.1 mg/ml heparin (Sigma-Aldrich, USA), endothelial cell growth supplement (Sigma-Aldrich, USA) and 10% fetal bovine serum (GIBCO, USA) and 1% penicillin-streptomycin (GIBCO, USA). Cells were maintained at 37°C, 5% CO_2_ humidified incubator.

### Sample preparation for microarray study

HUVEC (6 x 10^5^ cells) were seeded in 75 cm^2^ culture flask with complete medium. After 24 h of incubation, the cells were starved in medium with 0.5% of FBS for 24 h. The medium was replaced with fresh medium in the absence (control HUVEC) or presence of 75 μg/ml NF3 (NF3-treated HUVEC). After 6 h and 16 h incubation, cells were collected with addition of Trizol reagent and stored at -80°C for further RNA extraction.

### RNA isolation

Total cellular RNA was extracted by TRIzol® reagent (Invitrogen, USA) according to the manufacturer’s instructions. Freshly extracted RNA was measured by using a NanoDrop™ 2000 UV-visible spectrophotometer (Thermo Scientific, USA). RNA integrity was additionally assessed by using a Eukaryote total RNA nano chip and the Agilent 2100 Bioanalyser (Agilent Technologies, Germany).

### Microarray hybridization

cRNA was transcribed *in vitro* by incorporating a biotinylated pseudouridine molecule by using the Ambion® WT expression kit (Applied Biosystem, USA) and Affymetrix® reagent kits (Affymetrix, USA). Hybridization was performed by using GeneChip Human Gene 1.0 ST Array (Affymetrix, USA) containing 764,885 distinct probes corresponding to 28,869 well-annotated genes. After washing, the chips were stained with streptavidin–phycoerythrin in accordance with the Affymetrix EukGE-WS2v5 protocol by using a Fluidic FS450 station. The microarrays were read with the GeneChip Scanner 3000 (Affymetrix, USA). The Affymetrix GeneChip Operating Software version 1.2 was used to manage the Affymetrix GeneChip array data and to automate the control of the GeneChip fluidics stations and scanners.

### Analysis for genomic study

The acquisition and initial quantification of array images were conducted using the AGCC software (Affymetrix, USA). Subsequent data analyses were performed using Partek Genomics Suite Version 6.4 (Partek, USA). We firstly performed a one-way ANOVA to identify the genes between the groups at a P value of less than 0.05, and then calculated the relative difference in fold change between the groups. Although these criteria were used with low stringency, this was expected to detect more genes truly associated with NF3 treatment at the expense of increasing the number of false-positives to be validated by bio-informatics and qRT-PCR experimental means. Cluster analyses and principal component analysis (PCA) were conducted with Partek default settings.

### Bioinformatic analysis

The gene lists were further analyzed by Genomatix Pathway System (GePS; http://www.genomatix.de/index.html) which gave all canonical pathways, interactions and network construction with significant enrichment of the input proteins based on data from the NCI-Nature Pathway Interaction Database, Biocarta, etc. The selection parameter of the differentially expressed genes of molecular functions classification and connectivity network identification was based on the gene enrichment analysis with p < 0.05. The Universal Protein Resource (UniProt; http://www.uniprot.org/) was a comprehensive resource for annotating and identifying genes with respective to the gene ontology (GO), pathway and protein families from the input of gene list, providing a platform for the understanding of molecular functions and signaling pathways involved with the regulatory genes.

### Detection of mRNA expression level by quantitative reverse transcription real-time polymerase chain reaction (qRT-PCR)

The preparation and isolation of RNA were previously described. HUVEC were used in the quantitative reverse transcriptase real-time polymerase chain reaction (qRT-PCR) for confirmation of the selected genes. qRT-PCR was performed by using iScript™ one-step RT-PCR kit with SYBR® Green (Bio-Rad, USA) on CFX96 Real-Time PCR Detection System (Bio-Rad, USA) in accordance to the manufacturer’s instructions. Specific primers for target and reference genes were designed using OligoPerfectTM Designer (Life technologies, USA) or PrimerQuest (Integrated DNA technologies, USA). The primer sets for 6 h and 16 h time points were listed in Tables 1 and 2, respectively. Gene amplification was carried out in 10 μl reaction with the following program: 10 min of cDNA synthesis at 50°C; 5 min of initial denaturation at 95°C; 49 cycles of 10 s of denaturation at 95°C and 30 s of annealing and extension at 60°C. After amplification, a melting curve analysis from 65 to 95°C with heating rate of 0.5°C per 5 s with a continuous fluorescence acquisition was carried out. The experiments were repeated three times each. Relative expression of the qRT-PCR product was calculated by using the comparative 2^–ΔΔCt^ method. The gene expression level was determined by normalizing to the reference gene GAPDH mRNA level. The fold difference of NF3 treatment group was then determined by normalizing all values to the mean of the relative expression in control group.

**Table 1:** Primer list of NF3-treated HUVEC versus control at 6 h.

	Primer sequence (5'→3')	
Gene	Forward	Reverse
BIRC5	TCATAGAGCTGCAGGGTGGATTGT	AGTAGGGTCCACAGCAGTGTTTGA
CXCR7	ACGTGGTGGTCTTCCTTGTC	AGGGATGTAGTGCAGGATGG
DNAJB7	TCAGAATTTCACGACAGTCCGGGT	ACGTACCACCACACCCAGCTAAAT
EPCAM	AAGCTGGCCGTAAACTGCTTTGTG	GCAGCCAGCTTTGAGCAAATGACA
GAPDH	ACAGTCAGCCGCATCTTCTT	ACGACCAAATCCGTTGACTC
GUCY2C	TGGTGGCTAGTGGTTTGCCTAAGA	AGGAAGATGCTCCAGCTCAAAGGT
HAND1	ATTAACAGCGCATTCGCGGAGTTG	ATCCGCCTTCTTGAGTTCAGCCTT
HLA-DQB1	ATGACCCTGAAAGCAGACATCCCA	AGAAGGAAGAGCCTGCCAAAGACT
INSL4	AGCCTGTTCCGGTCCTATCT	CAGCTCTGCTGCTAGGCTTT
MAP3K14	TGCGGAAAGTGGGAGATCCTGAAT	TGTACTGTTTGGACCCAGCGATGA
MMP8	AATCCTTGCTCATGCCTTTCAGCC	GTGTTGGTCCATGTTTCTTCGGCA
NPR3	TGGCGATGAAGGTGGTAGTTCACA	TTCTCCAGGGAAGCACTCACACAT
PGC	ATGATCCTGCTTGGAAGTACCGCT	TCTCACCAAAGTAGGCAGCATCCA
SLC5A8	GGTTGGTGGACCACTTATGG	AATCCAGCCATCAGACCAAC
UBD	TGATGAGGAGCTGCCCTTGTTTCT	TTGCTTTCACTTGTGCCACTGAGC

**Table 2:** Primer list of NF3-treated HUVEC versus control at 16 h.

	Primer sequence (5'→3')	
Gene	Forward	Reverse
ABCG8	TGTCAATGACCATCGGCTTCCTCT	ATCATGAACAAGAGGGCGGCTGTA
EPB41	TCCCAAAGTGCTGGGATTACAGGT	AGCCTTGAGGCCAATGTGTTTGTG
GAPDH	ACAGTCAGCCGCATCTTCTT	ACGACCAAATCCGTTGACTC
GRIN1	GTCTCCCTGTCCATCCTCAA	CTGATACCGAACCCACGTCT
GYPA	GCACTCACTGAAGCTTGCATTCCA	TGCATGTCCGGTTTGCACATCTTG
IL1RAPL2	AACCGAAATGGACGGAAACATGCC	AGCTCCAAAGTGCTGCCTGTAGAA
LCN1	CCGTCCTGGAGAAAACTGAC	ACGTGCGACCTGATGATGTA
LRFN2	TCAGGGAATGTGGACTCGCTCAAA	AATCCAAAGCCGCTTGCTTGTAGG
MIR28	CCCTCAAGGAGCTCACAGTC	CCTCCAGGAGCTCACAATCT
NR1H4	AACAGAACAAGTGGCAGGTCCTCT	AGCTGGCATACGCCTGAGTTCATA
PRL	TGGCTGATGAAGAGTCTCGCCTTT	TTCGGCACTTCAGGAGCTTGAGAT
SCG2	AGGGATGGAGAGTGCAGCAAATCA	AGCTTTGGGAAGCTGGTTCGATCT
TFAP2A	GGGTACGTGTGCGAAACCGAATTT	TGTCACTTGCTCATTGGGATCGGA
TTC39B	ATGTACCATGCCTTGGGCTACAGT	GTCCTTCATGGCAGAAATGCCGTT
ZNF711	TTCGTGGCTGACCTTGTTACTGGT	ACCTCCAGCTGCTTGAATCACAGT

### Protein extraction and western blotting

The NF3-treated cells at 6 and 16 h were collected and lysed in a RIPA buffer for 30 min on ice, and then centrifuged at 14000 rpm for 15 min at 4°C. Protein concentration was measured using Bio-Rad Dc Protein Assay (Bio-Rad, Hercules, CA USA). Equal amount (40 μg) of protein samples were separated on a 8% SDS-polyacrylamide gel and electrophoretically transferred (100 V, 2 h) onto a nitrocellulose membrane (Pall Gelman Laboratory, Ann Arbor, MI USA). Afterwards the membranes were blocked for 1 h using 5% non-fat dry milk, and then incubated overnight at 4°C with primary antibodies, including CXCR7 (GTX100027; GeneTex, USA), MMP8 (ab81286; Abcam, USA), NR1H4 (ab28676; Abcam, USA) and SCG2 (26101; QED Bioscience, USA). After washing the membranes, membranes were incubated with the secondary horseradish peroxidase-conjugated antibodies (Invitrogen, Carlsbad, CA, USA) for 1 h, detection was performed using enhanced chemiluminescence assay kit (GE Healthcare, UK).

### Statistical analysis

The differences in the gene expression level between the NF3-treated and the control group were compared by a Student’s t test with p < 0.05 as considered statistically significant. All data were expressed as mean ± standard deviation (SD). Statistical analyses were carried out using GraphPad Prism version 5.00 for Windows (GraphPad software, USA).

## Results

### Comparison of gene expression profile by principal component analysis (PCA)

**Fig 1:**
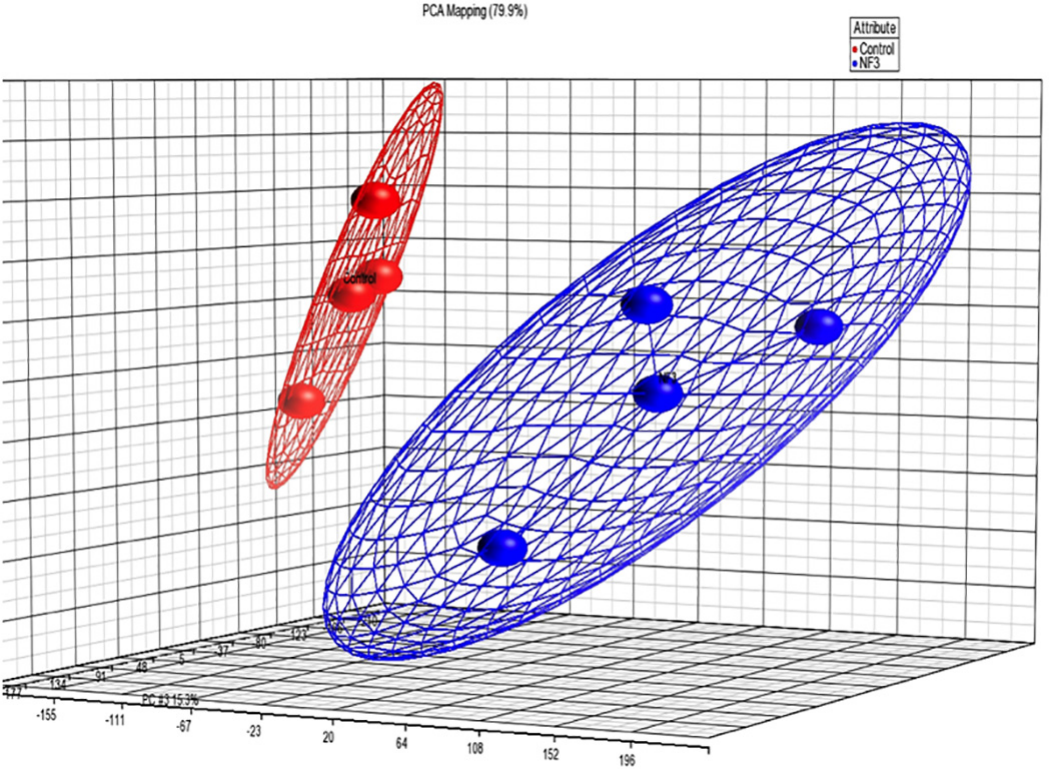
Supervised principal component analysis (PCA). Microarray hybridizations were performed using total RNA from HUVEC treated with 75 μg/ml NF3 for 16 h. The gene expression profiles of 3 pools of control cells (red) and 3 pools of NF3-treated cells (blue) were compared using PCA. The red or blue ball located at the central position within the cluster represented the mean of gene expression profiles of 3 pools of control or NF3-treated cells. The two distinct clusters of three red and blue balls represented the intra-experimental variation of control cells and NF3-treated cells, respectively. The three-dimensional plot view of gene expression data (including all probe sets on human GeneChip 1.0 ST) were shown with respect to their correlation to the first three principal components.

Principal component analysis (PCA) is a statistical technique for determining the key variables in a multidimensional data set. It clearly identifies the inter-experimental variations of the differential expression pattern of two groups associated with the validation of intra-experimental variations of the microarray experiment. The two distinct clusters of three red and blue balls represented the intra-experimental variation of control cells and NF3-treated cells, respectively. The PCA results illustrated that there was no overlap in the clusters of three repeated hybridizations (balls in the same color) of the control HUVEC (red) and NF3-treated HUVEC (blue) (Fig 1). The gene expression profile of NF3-treated HUVEC group was well separated from that of control HUVEC group.

### Identification of global gene expression changes in 6 h and 16 h of NF3-treated HUVEC

The pro-angiogenic activities of HUVEC with different concentrations of NF3 were previously demonstrated [5]. A significant cell migration and tube formation effect were found in HUVEC treated with 75 μg/ml NF3. For comprehensive microarray study, 75 μg/ml concentration of NF3 was used for global genomic expression analysis in HUVEC for 6 h and 16 h incubation. The raw data generated by AGCC software were further processed by Partek Genomics Suite, which identified the genes between NF3-treated HUVEC and control HUVEC with criteria settings of fold change above 1.2 and p value lower than 0.05. Although these criteria were used with low stringency, more genes associated with NF3 treatment could be detected. The number of false-positive results would be validated further by bio-informatics analysis and qRT-PCR. The relative gene expression difference was presented as the fold change of NF3-treated HUVEC versus control HUVEC. From the Partek software analysis, 103 genes were identified with 61 up-regulated and 42 down-regulated in NF3-treated HUVEC versus control HUVEC at 6 h. The panels of differentially expressed genes with accession number, gene symbol, gene name and p value were shown in S1 Table. At 16 h, we found a total of 96 genes with 32 up-regulated and 64 down-regulated in NF3-treated HUVEC versus control HUVEC, which were listed in S2 Table.

### Functional categorization of genes

**Fig 2:**
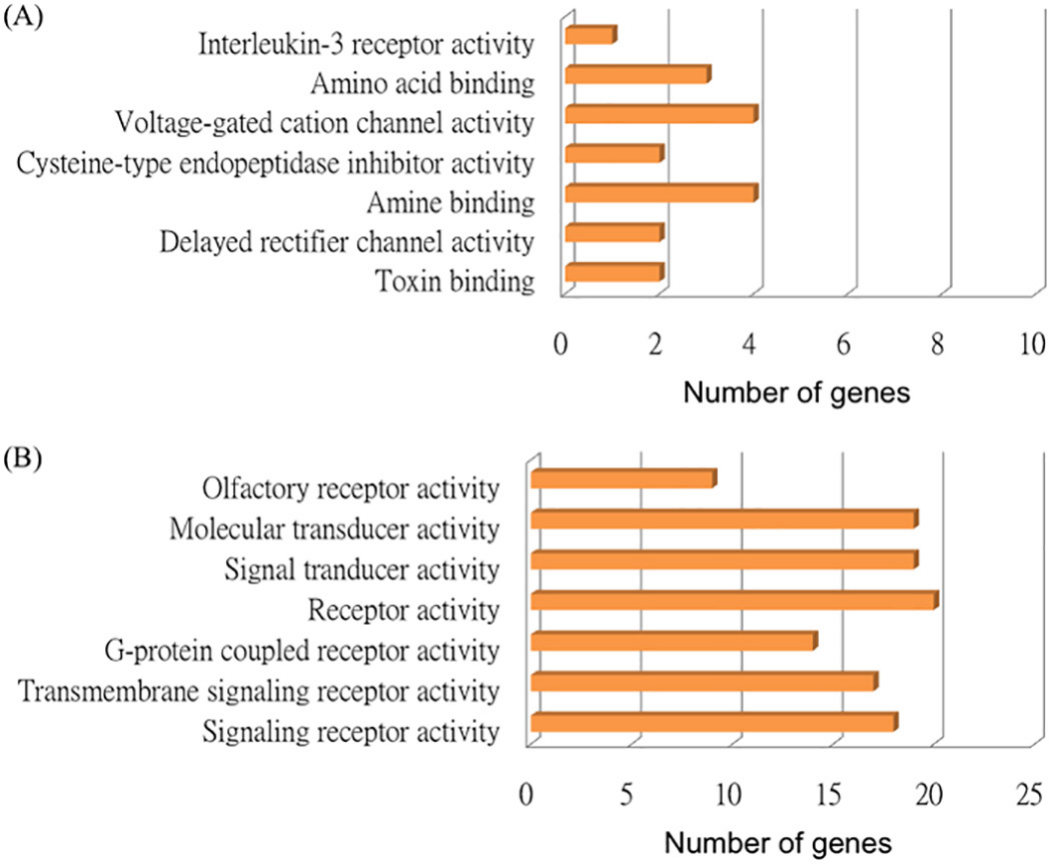
The molecular functions of differentially expressed genes in HUVEC after (A) 6 h and (B) 16 h of NF3 treatment.

Genomatix Pathway System (GePS) was applied to classify the differentially expressed genes into molecular functions and biological processes based on an AmiGo gene ontology (GO) database with significance of p < 0.05. As shown in Fig 2A, the most significant molecular function of NF3-treated HUVEC versus control HUVEC at 6 h was toxin binding consisting of GUCY2C and HLA-DQB1 (p = 8.3 x 10^−4^). Delayed rectifier channel activity included KCNE1 and KCNH1 (p = 1.21 x 10^−4^). However, only a few genes were grouped into molecular functions with significances from the gene list of NF3-treated HUVEC versus control HUVEC at 6 h. Not much molecular information could be obtained. Surprisingly, more genes in NF3-treated HUVEC versus control HUVEC at 16 h were grouped into different significant molecular functions (Fig 2B). 18 genes were classified into signaling receptor activity (p = 1 x 10^−5^), while 17 genes belonged to transmembrane signaling receptor activity (p = 1.55 x 10^−5^). Besides, a number of genes had G-protein coupled receptor activity, signal tranducer activity and receptor activity. The molecular function categorization provided molecular details among the genes, postulating the specific regulation of different panel of differentially expressed genes in NF3-treated HUVEC versus control HUVEC at different time frame.

### Network connectivity identification

**Fig 3:**
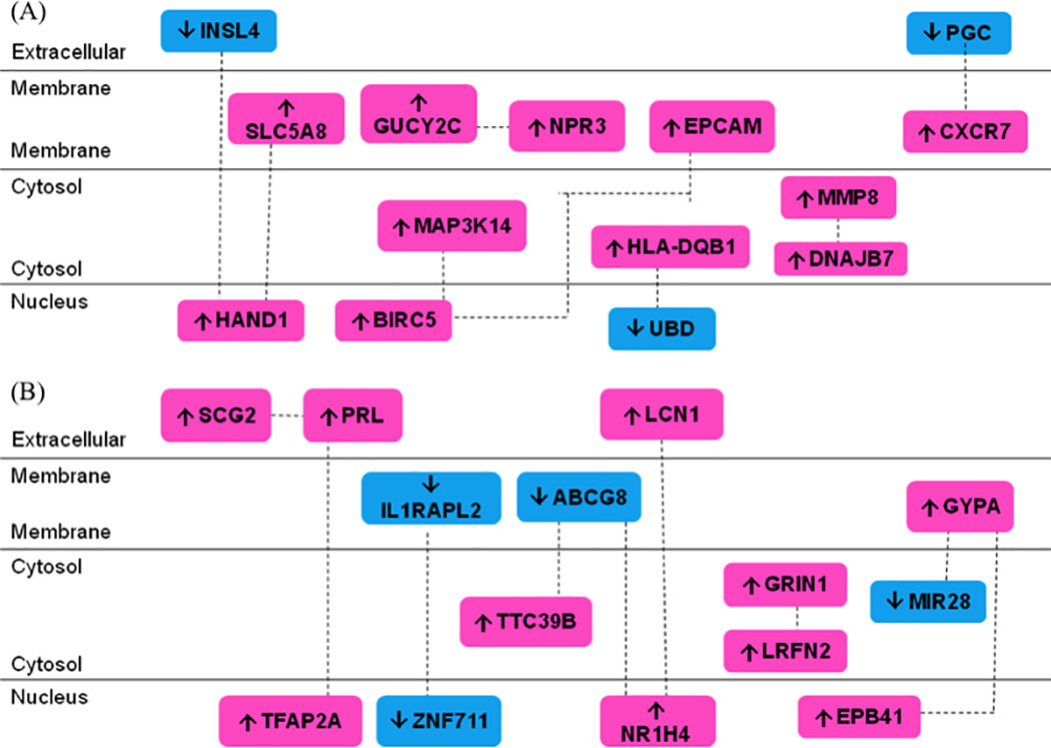
Network connectivity of identified genes in NF3-treated HUVEC by GePS. (A) Differentially expressed genes with frequent co-citations and interactions in NF3 treated HUVEC at 6 h. (B) Differentially expressed genes with frequent co-citations and interactions in NF3 treated HUVEC at 16 h. Genes were identified by their gene symbols (S1 and S2 Tables). Dashed lines indicated association by co-citation. Up arrow and down arrow corresponded to up- or down-regulation, respectively.

To elucidate the regulatory mechanisms of NF3 on the differentially expressed genes of HUVEC involved in wound healing angiogenesis, the genes were annotated by GePS according to their relative interactions and frequent co-citation from data and literature mining so as to predict their biological processes and underlying signaling pathways. The connectivity map organized genes based on their interactions, which could further extend the input genes by transcription factors or transcriptional downstream targets. In Fig 3, genes with differential expression in HUVEC after 6 h and 16 h of NF3 treatment were linked in the connectivity network. The advantage of connectivity map by GePS was to sort out genes with interactions for subsequent pathway prediction. All the target genes were further analyzed using the Universal Protein Resource (UniProt) in order to categorize them according to gene ontology (GO) and respective protein families. As illustrated in Table 3 of HUVEC after 6 h of NF3 treatment, BIRC5 was annotated to regulate cell cycle and migration behavior (microtubule organizing center). CXCR7 and HAND1 were responsible for neovascularization of endothelial cells. Some genes (DNAJB7 and HAND1) possessed DNA binding transcription factor activity. EPCAM, GUCY2C and INSL4 were directly involved in cell proliferation. Genes with protease activity participating in different cellular reaction included MAP3K14, MMP8 and PGC. On the other hand, after 16 h of NF3 treatment (Table 4), different genes were triggered and expressed in HUVEC. EPB41 was identified to participate in actin cytoskeleton organization, governing cell motility. IL1RAPL2 contained interleukin-1 receptor activity, transmitting inflammatory signal of interleukin-1. SCG2 was the candidate gene responsible for angiogenesis as well as regulation of proliferation and migration of endothelial cells. NR1H4, TFAP2A and ZNF711 belonged to DNA binding transcription factor activity or regulation of transcription. From the GO analysis, the differentially expressed genes in HUVEC would provide insight of the regulatory mechanisms as well as the prediction of the signaling pathways elicited by NF3 on mature endothelial cells in modulation of wound healing angiogenesis.

**Table 3:** Gene ontology classification of the identified genes in NF3-treated HUVEC at 6 h by UniProt.

Gene	Protein names	Gene ontology (GO)	Protein families
BIRC5	Baculoviral IAP repeat-containing protein 5	G2/M transition of mitotic cell cycle; microtubule organizing center	IAP family
CXCR7	C-X-C chemokine receptor type 7	Vasculogenesis; angiogenesis; cell adhesion	G-protein coupled receptor 1 family
DNAJB7	DnaJ homolog subfamily B member 7	DNA binding transcription factor activity	—
EPCAM	Epithelial cell adhesion molecule	Positive regulation of cell proliferation	EPCAM family
GUCY2C	Heat-stable enterotoxin receptor	Intracellular signal transduction; regulation of cell proliferation	Adenylyl cyclase class-4/guanylyl cyclase family
HAND1	Heart- and neural crest derivatives-expressed protein 1	Angiogenesis; embryonic heart tube formation; mesoderm formation; DNA binding transcription factor activity	—
HLA- DQB1	major histocompatibility complex, class II, DQ beta 1; similar to major histocompatibility complex, class II, DQ beta 1	—	—
INSL4	Early placenta insulin-like peptide	Cell proliferation; cell-cell signaling; insulin-like growth factor receptor binding	Insulin family
MAP3K14	Mitogen-activated protein kinase kinase kinase 14	MAPK cascade; NF-kappab-inducing kinase activity; T cell costimulation; activation of MAPKK activity	Protein kinase superfamily
MMP8	Matrix metalloproteinase-8	Collagen catabolic process; extracellular matrix disassembly	Peptidase M10A family
NPR3	Natriuretic peptide receptor C	Natriuretic peptide receptor activity	—
PGC	Pepsinogen C	Aspartic-type endopeptidase activity; proteolysis	Peptidase A1 family
SLC5A8	Sodium-coupled monocarboxylate transporter 1	Sodium ion transport; symporter activity; transmembrane transport	Sodium:solute symporter (SSF) (TC 2.A.21) family
UBD	Ubiquitin D	Positive regulation of I-kappab kinase/NF-kappab cascade; positive regulation of apoptotic process; proteasome binding; protein ubiquitination	—

**Table 4:** Gene ontology classification of the identified genes in NF3-treated HUVEC at 16 h by UniProt.

Gene	Protein names	Gene ontology (GO)	Protein families
ABCG8	ATP-binding cassette sub-family G member 8	ATP catabolic process; cholesterol homeostasis	ABC transportersuperfamily
EPB41	EPB41 protein	Actin cytoskeleton organization	—
GRIN1	Glutamate receptor ionotropic, NMDA 1	—	Glutamate-gated ion channel (TC 1.A.10.1) family
GYPA	Glycophorin-A	Cytoskeletal anchoring at plasma membrane; response to osmotic stress	Glycophorin A family
IL1RAPL2	Interleukin 1 receptor accessory protein-like 2	Interleukin-1 receptor activity	Interleukin-1 receptor family
LCN1	Lipocalin-1	Cysteine-type endopeptidase inhibitor activity	Calycin superfamily
LRFN2	Leucine rich repeat and fibronectin type III domain containing 2	Cell junction	LRFN family
MIR28	MicroRNA 28	—	—
NR1H4	Nuclear receptor subfamily 1, group H, member 4	DNA binding transcription factor activity; positive regulation of transcription	Nuclear hormone receptor family
PRL	Prolactin	JAK-STAT cascade involved in growth hormone signaling pathway; cell proliferation	Somatotropin/ prolactin family
SCG2	Secretogranin-2	Angiogenesis; regulation of endothelial cell proliferation and migration; chemoattractant activity; cytokine activity	Chromogranin/ secretogranin protein family
TFAP2A	Transcription factor AP-2-alpha	DNA binding transcription factor activity involved in positive regulation of transcription	AP-2 family
TTC39B	Tetratricopeptide repeat protein 39B	—	TTC39 family
ZNF711	Zinc finger protein 711	Positive regulation of transcription	Krueppel C2H2-type zinc-finger protein family

### Microarray analysis and data verification by qRT-PCR

qRT-PCR was employed to validate the expression level of the selected genes from microarray analysis. We determined the mean value of expression of the selected genes in 3 independent NF3-treated HUVEC samples at 6 h and 16 h. The qualitative changes in gene expression levels were consistent between the analyses. Quantitative difference was greater in qRT-PCR than in microarray analysis. The expression levels of the target genes in NF3-treated HUVEC versus control HUVEC at 6 and 16 h were listed in Tables 5 and 6, respectively.

**Table 5:** qRT-PCR analysis of the relative gene expression level of NF3-treated HUVEC versus control HUVEC at 6 h. RNA from new preparation of HUVEC samples were used for confirmation of microarray analysis.

	Fold change		
	NF3	Control	P-value
BIRC5	1.61	1.42	0.003
CXCR7	1.93	0.86	0.039
DNAJB7	0.82	0.66	0.044
EPCAM	1.43	1.18	0.027
GUCY2C	2.62	2.16	0.216
HAND1	0.74	0.48	0.043
HLA-DQB1	0.83	0.70	0.235
INSL4	0.44	0.64	0.013
MAP3K14	0.56	0.67	0.014
MMP8	0.76	0.45	0.028
NPR3	1.21	0.80	0.033
PGC	0.79	0.87	0.191
SLC5A8	0.46	0.30	0.005
UBD	0.35	0.52	0.016

Values are means ± SD from three individual experiments.

**Table 6:** qRT-PCR analysis of the relative gene expression level of NF3-treated HUVEC versus control HUVEC at 16 h. RNA from new preparation of HUVEC samples were used for confirmation of microarray analysis.

	Fold change		
	NF3	Control	P-value
ABCG8	0.58	0.70	0.568
EPB41	1.35	0.74	0.001
GRIN1	1.20	2.65	0.036
GYPA	1.70	0.98	0.078
IL1RAPL2	0.80	1.44	<0.001
LCN1	2.46	1.32	0.027
LRFN2	2.48	1.87	0.107
MIR28	1.08	1.86	0.019
NR1H4	0.79	0.41	0.032
PRL	2.52	1.18	0.004
SCG2	0.74	0.43	0.002
TFAP2A	1.02	0.47	0.006
TTC39B	1.48	1.32	0.296
ZNF711	1.45	1.62	0.387

Values are means ± SD from three individual experiments.

### Protein expression of the target genes by western blotting

**Fig 4:**
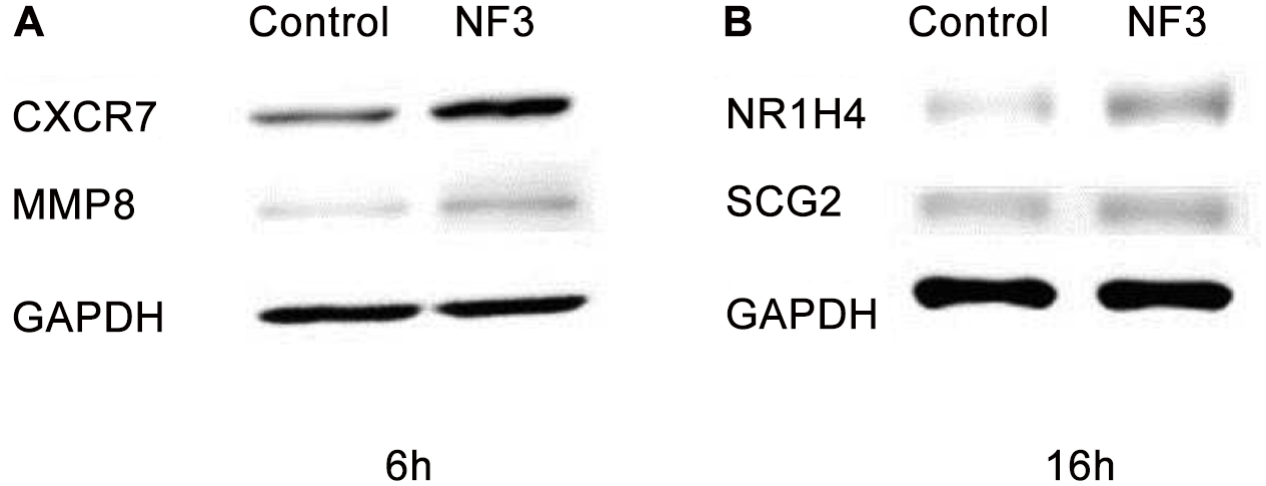
Western blotting of NF3 on HUVEC for 6 and 16 h. Immunobloting was performed three times using independent cell lysates and representative blots of (A) 6 h and (B) 16 h were shown.

Western blotting of the protein expression was conducted so as to confirm the differentially expressed gene targets revealed from microarray data. Since a panel of different sets of genes was believed to be involved in HUVEC after NF3 treatment for 6 and 16 h, two representative proteins at each time point were selected for further confirmation. The selection criteria of the proteins from these sets of genes were based on the role of the genes and the availability of antibody for western blotting. CXCR7 and MMP8 were used at 6 h while NR1H4 and SCG2 were examined at 16 h. As shown in Fig 4, NF3 caused a strong induction of the protein expression of CXCR7 in HUVEC associated with an increase in the protein expression of MMP8 and NR1H4 in HUVEC. A mild increase in the protein expression of SCG2 was observed in NF3-treated HUVEC. The results demonstrated that both genes and proteins were tightly involved and regulated in HUVEC after NF3 treatment, promoting the overall wound healing angiogenesis.

## Discussion

The individual herb AR and RR had been historically used in diabetic foot ulcer healing. Increasing studies provided concrete scientific proof for the diabetic wound healing efficacy and pro-angiogenic capacity of formula NF3 [5–9].

The merit of Traditional Chinese Medicine (TCM) was its holistic pharmaceutical characteristic with multi-target approach, improving the whole biological system and making TCM as promising alternative therapies in different diseases. Substantial *in vitro* and clinical studies demonstrated the involvement of diverse signaling pathways of NF3 involved in diabetic wound healing. Zhang *et al*. (2012) showed that NF3 triggered a panel of genes in human skin fibroblast involved in various signaling pathways in wound healing [12]. Ko *et al*. (2014) further showed that NF3 was effective in healing of diabetic foot ulcer patients associated with the global changes in gene expression [13]. We therefore applied microarray technology to screen out the regulatory genes in endothelial cells with NF3 intervention in relation to angiogenesis and diabetic wound healing.

Endothelial cells activation followed by diverse intracellular signaling pathways of proliferation, adhesion, migration, tubular formation, release of growth factors and matrix metalloproteinases enabled proper angiogenesis in wound healing process. However, in diabetic ulcers, the angiogenesis was hampered by persistent hyperglycemic condition, causing destructive effects on cellular and molecular levels in endothelial cells.

**Fig 5:**
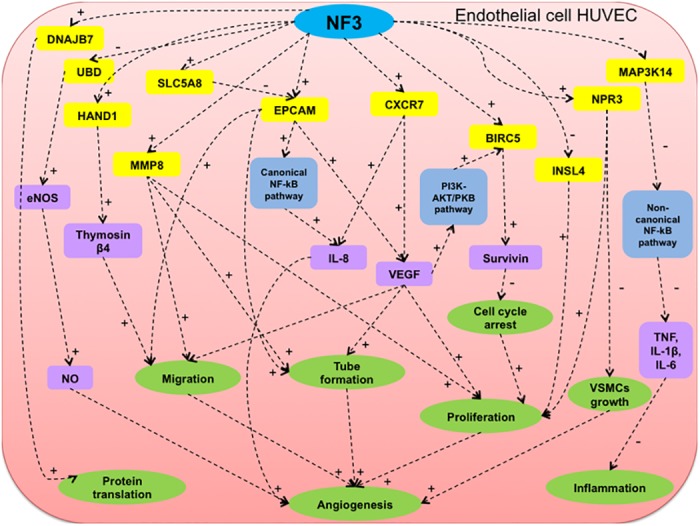
Hypothetical diagram of mechanistic actions of NF3 on HUVEC at 6 h. The molecular gene targets, respective signaling pathways and molecule mediators associated with the biological responses was demonstrated in NF3-treated HUVEC at 6 h. Yellow molecules represented differentially expressed genes identified from microarray analysis. Blue box represented the suggested signaling pathway. Purple box represented the molecular mediators. Green box represented the biological responses. + indicated as positive regulation and–indicated negative regulation.

In the present study, we investigated the effect of NF3 treatment on global gene expression of HUVEC governing angiogenesis in wound healing after 6 h and 16 h. From the microarray analysis associated with qRT-PCR and western blotting verification, target genes (BIRC5, CXCR7, DNAJB7, EPCAM, HAND1, MMP8, NPR3 and SLC5A8) were significantly up-regulated, while target genes (INSL4, MAP3K14 and UBD) were significantly down-regulated in NF3-treated HUVEC when compared to that of control HUVEC at 6 h. BIRC5 gene encoded the protein survivin, which regulated the apoptosis pathway. BIRC5 was crucial for optimal angiogenesis after brain injury in VEGF-treated HUVEC [14]. CXCR7, a member of CXC chemokine receptor family, possessed high binding affinity to chemokine stromal derived factor-1α (SDF-1α). Up-regulation of CXCR7 was vitally important in angiogenesis, stimulating the production of interleukin-8 (IL-8) and VEGF [15–17]. The protein of DNAJB7 gene belonged to the DNAJ/HSP40 family, which regulated molecular chaperone activity [18]. EPCAM mediated cell-cell adhesion, migration, proliferation and differentiation. EPCAM was asserted to possess oncogenic potential and up-regulation of EPCAM induced angiogenesis [19–21]. The protein encoded by HAND1 was classified into the basic helix-loop-helix family of transcription factors. Recent finding identified that thymosin β4 was an *in vivo* downstream target of HAND1, which was crucial for cell migration and angiogenesis as well as yolk sac vasculogenesis [22, 23]. The functional involvement of MMP8 (matrix metalloproteinase 8) in angiogenesis and tissue remodeling was extensively investigated [24, 25]. Up-regulation of NPR3 (natriuretic peptide receptor 3) activated ECs proliferation and meanwhile inhibited vascular smooth muscle cells (VSMCs) growth [26, 27]. SLC5A8 was identified as one of the tumor suppressor genes [28, 29]. For those down-regulated genes, INSL4 (insulin-like 4 protein) was the member of insulin superfamily. It was suggested that down-regulation of INSL4 might contribute to the cell survival and anti-apoptotic ability [30, 31]. MAP3K14 (mitogen-activated protein kinase kinase kinase 14) was a serine/threonine protein-kinase. Up-regulation of MAP3K14 had been linked to the persistent inflammatory response in diabetes [32, 33]. UBD was one of the components in the ubiquitin-proteasome system (UPS). It had been found that inhibition of UPS up-regulated endothelial nitric oxide synthase (eNOS) expression and activity, thus improving the endothelial function [34, 35]. By summarizing the biological function among the differentially expressed genes in NF3-treated HUVEC at 6 h, their respective roles were postulated, suggesting their beneficial effects on diverse mechanisms related to migration, tube formation, proliferation, angiogenesis as well as anti-inflammation in HUVEC (Fig 5).

**Fig 6:**
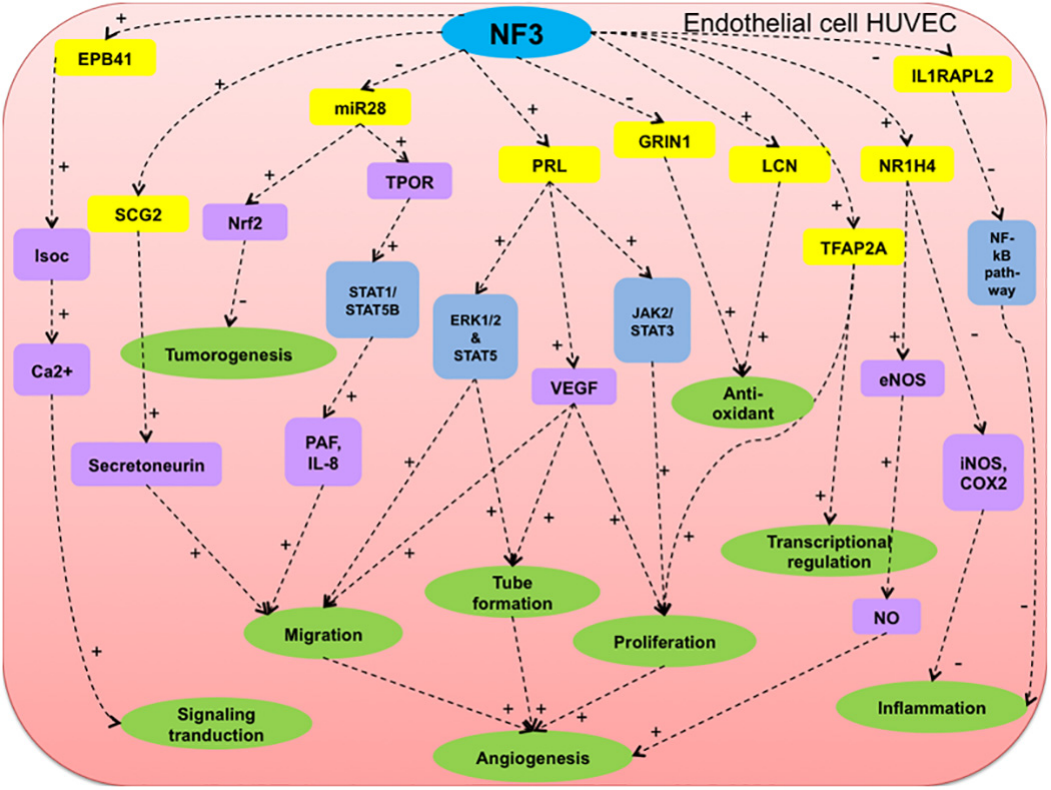
Hypothetical diagram of mechanistic actions of NF3 on HUVEC at 16 h. The molecular gene targets, respective signaling pathways and molecule mediators associated with the biological responses was demonstrated in NF3-treated HUVEC at 16 h. Yellow molecule represented differentially expressed genes identified from microarray analysis. Blue box represented the suggested signaling pathway. Purple box represented the molecular mediators. Green box represented the biological responses. + indicated as positive regulation and–indicated negative regulation.

As demonstrated by the gene expression results of NF3-treated HUVEC versus control HUVEC at 16 h, genes (EPB41, LCN1, NR1H4, PRL, SCG2 and TFAP2A) were significantly up-regulated while genes (GRIN1, IL1RAPL2 and miR28) were significantly down-regulated. Among those up-regulated genes of NF3-treated HUVEC at 16 h, protein 4.1 encoded by EPB41 gene was required for the regulation of channel activation and ion permeation for signaling transduction in ECs [36, 37]. LCN1 gene encoded a member of the lipocalin family of small secretory proteins. LCN1 was devoted to the cellular defense against oxidative stress [38]. NR1H4 (protein farnesoid X receptor (FXR)) was a member of the nuclear receptor superfamily. FXR was confirmed to induce eNOS expression at transcriptional level since up-regulation and subsequent NO production [39]. FXR activation in turn down-regulated inducible nitric oxide synthase (iNOS) and cyclooxygenase-2 (COX-2), which were the pro-inflammatory mediators. FXR signaling pathway played a pivotal role in vascular anti-inflammation and remodeling [40]. Substantial reports demonstrated the advantageous role of PRL (prolactin) in angiogenesis [41–43]. SCG2 gene encoded the protein secretogranin, which was converted to chemotactic active secretoneurin. Secretoneurin was involved in inflammation and wound healing [44]. Secretoneurin significantly induced the chemotaxis ability of ECs, confirming the regulatory role of secretoneurin in vascular cells migration [45]. The protein named transcription factor activator protein-2A (AP-2A) was encoded by TFAP2A gene, which was involved in transcriptional regulation and cell proliferation [46, 47]. For those down-regulated genes, study revealed that GRIN1 (N-methyl-D-aspartate receptor (NMDAR)) activation in human cerebral ECs promoted intracellular oxidative stress [48]. IL1RAPL2 (interleukin-1 receptor accessory protein-like 2) was the member of interleukin-1 receptor (IL-1R) family for signaling transduction of pro-inflammatory cytokines within cells [49]. miR28 was a member of the small non-coding RNAs called microRNAs (miRNAs). Elevation of endothelial miR28 impaired angiogenesis in diabetic patients [50]. Nrf2 regulated the expression of detoxifying enzymes, protecting cells towards tumorogenesis. Up-regulation of miR28 inhibited Nrf2 mRNA and protein expression, leading to breast cancer progression [51]. Besides, miR28 inhibited the translation of thrombopoietin receptor (TPOR). Activation of TPOR in HUVEC demonstrated enhanced cell motility through synthesis of 1-alkyl-/1-acyl-platelet-activating factor (PAF), IL-8, and phosphorylation of STAT1 and STAT5B [52, 53]. From numerous literature support, those differentially expressed genes at 16 h were proposed to devote pivotal role in wound healing angiogenesis, governing the diverse signaling pathways of transcriptional and translational regulation, anti-tumor activity, anti-oxidant activity, angiogenesis and anti-inflammation in NF3-treated HUVEC (Fig 6).

In summary, the molecular gene targets were identified in NF3-treated HUVEC and their expression was further confirmed by qRT-PCR. Different panels of genes were differentially expressed and strictly governed in NF3-treated HUVEC in a time-regulated manner. In the investigation of two time points, we showed that NF3 regulated different set of genes in HUVEC to achieve various biological processes such as ECs proliferation, migration, tube formation, transcriptional and translational regulation, anti-inflammation and anti-oxidation. This might explain the underlying mechanism of NF3-induced angiogenesis of endothelial cells involved in wound healing. Interestingly, the results revealed that different sets of genes were differentially expressed and regulated in HUVEC by NF3 in two time points. In general, there was a strong correlation between time series and gene expression in every cell. We had performed time series analysis of NF3-treated HUVEC in tube formation assay from 4 to 8 h and migration assay from 16 to 20 h (data not shown). NF3 significantly stimulated the tube formation and migration ability of HUVEC in the tested time points. In line with this, it was extremely important to choose the most appropriate time points for evaluating the global gene expression pattern changes in HUVEC after NF3 treatment. The tubular formation at 6 h and migration at 16 h of NF3 on HUVEC were shown to have profound significant pro-angiogenic effects [5]. Therefore 6 h and 16 h incubation of NF3 in HUVEC were selected for microarray analysis.

With reference to the assessment of molecular targets of HUVEC after NF3 treatment by microarray technology, we constructed novel hypothetical diagrams to illustrate the regulatory mechanism and signaling pathways of NF3 on endothelial cells involved in wound healing angiogenesis at transcriptional levels. The identified gene and targets enabled us to thoroughly understand the therapeutic efficacies of NF3 in diabetic wound healing angiogenesis. This study provided valuable genetic database with support of scientific evidences for microarray research using TCM on endothelial cells in different diseases.

## Supporting Information

S1 Table Differentially expressed genes in NF3-treated HUVEC versus control at 6 h from microarray analysis. (DOCX)

S2 Table Differentially expressed genes in NF3-treated HUVEC versus control at 16 h from microarray analysis. (DOCX)

S1 Raw Data (XLSX)

S2 Raw Data (XLSX)
