# Supplementary material for: Identification of Target Genes Involved in Wound Healing Angiogenesis of Endothelial Cells with the Treatment of a Chinese 2-Herb Formula
Source: PLoS One. 2015 Oct 2;10(10):e0139342. doi: 10.1371/journal.pone.0139342 (PMC4591983; doi:10.1371/journal.pone.0139342)
Supplement: S1 Table — (DOCX) [file pone.0139342.s001.docx]

**S1 table. Differentially expressed genes in NF3-treated HUVEC versus control at 6 h from microarray analysis.**

| Accession Number | Gene Symbol | Gene Name | P Value |
| --- | --- | --- | --- |
| NM_138340 | ABHD3 | Abhydrolase domain containing 3 | 0.019 |
| NM_024783 | AGBL2 | ATP/GTP binding protein-like 2 | 0.003 |
| NM_001145128 | AKD1 | Adenylate kinase domain containing 1 | 0.005 |
| NM_001168221 | ALS2CR11 | Amyotrophic lateral sclerosis 2 (juvenile) chromosome region, candidate 11 | 0.046 |
| NR_026844 | ANKRD36BP1 | Ankyrin repeat domain 36B-like 1 (pseudogene) | 0.029 |
| NM_015230 | ARAP2 | Arfgap with rhogap domain, ankyrin repeat and PH domain 2 | 0.019 |
| NM_016116 | ASB4 | Ankyrin repeat and SOCS box-containing 4 | 0.026 |
| ENST  00000398868 | ATL3 | Atlastin gtpase 3 | 0.031 |
| NM_018842 | BAIAP2L1 | BAI1-associated protein 2-like 1 | 0.020 |
| NM_000055 | BCHE | Butyrylcholinesterase | 0.012 |
| NM_001168 | BIRC5 | Baculoviral IAP repeat-containing 5 | 0.045 |
| NM_025063 | C1orf129 | Chromosome 1 open reading frame 129 | 0.017 |
| NM_182517 | C1orf210 | Chromosome 1 open reading frame 210 | 0.049 |
| BC114945 | C20orf152 | Chromosome 20 open reading frame 152 | 0.029 |
| NR_024622 | C21orf49 | Chromosome 21 open reading frame 49 | 0.037 |
| NR_027252 | C2orf58 | Chromosome 2 open reading frame 58 | 0.042 |
| NM_020161 | C2orf83 | Chromosome 2 open reading frame 83 | 0.002 |
| NM_152571 | C9orf163 | Chromosome 9 open reading frame 163 | 0.015 |
| NR_023352 | C9orf95 | Chromosome 9 open reading frame 95 | 0.041 |
| NM_006078 | CACNG2 | Calcium channel, voltage-dependent, gamma subunit 2 | 0.039 |
| NM_005623 | CCL8 | Chemokine (C-C motif) ligand 8 | 0.019 |
| NM_030893 | CD1E | CD1e molecule | 0.018 |
| NM_001029954 | CDNF | Cerebral dopamine neurotrophic factor | 0.032 |
| NM_001079827 | CLRN2 | Clarin 2 | 0.015 |
| NM_020311 | CXCR7 | Chemokine (C-X-C motif) receptor 7 | 0.018 |
| NM_000782 | CYP24A1 | Cytochrome P450, family 24, subfamily A, polypeptide 1 | 0.008 |
| NM_006716 | DBF4 | DBF4 homolog (S. Cerevisiae) | 0.015 |
| NM_145174 | DNAJB7 | Dnaj (Hsp40) homolog, subfamily B, member 7 | 0.020 |
| NM_004413 | DPEP1 | Dipeptidase 1 (renal) | 0.010 |
| NM_001385 | DPYS | Dihydropyrimidinase | 0.004 |
| NM_002354 | EPCAM | Epithelial cell adhesion molecule | 0.001 |
| NM_178516 | EXOC3L | Exocyst complex component 3-like | 0.002 |
| NM_172366 | FBXO16 | F-box protein 16 | 0.034 |
| NM_022110 | FKBPL | FK506 binding protein like | 0.032 |
| NR_026835 | FLJ37201 | Tigger transposable element derived 2 pseudogene | 0.047 |
| NM_004477 | FRG1 | FSHD region gene 1 | 0.046 |
| NM_014728 | FRMPD4 | FERM and PDZ domain containing 4 | 0.010 |
| NM_004963 | GUCY2C | Guanylate cyclase 2C (heat stable enterotoxin receptor) | 0.000 |
| NM_004821 | HAND1 | Heart and neural crest derivatives expressed 1 | 0.019 |
| NM_018437 | HEMGN | Hemogen | 0.047 |
| AK097297 | HLA-DQB1 | Major histocompatibility complex, class II, DQ beta 1; similar to major histocompatibility complex, class II, DQ beta 1 | 0.026 |
| NM_002183 | IL3RA | Interleukin 3 receptor, alpha (low affinity) | 0.049 |
| NM_002195 | INSL4 | Insulin-like 4 (placenta) | 0.030 |
| NM_000219 | KCNE1 | Potassium voltage-gated channel, Isk-related family, member 1 | 0.045 |
| NM_133329 | KCNG3 | Potassium voltage-gated channel, subfamily G, member 3 | 0.045 |
| NM_172362 | KCNH1 | Potassium voltage-gated channel, subfamily H (eag-related), member 1 | 0.014 |
| NM_198689 | KRTAP10-7 | Keratin associated protein 10-7 | 0.001 |
| NM_181621 | KRTAP13-2 | Keratin associated protein 13-2 | 0.037 |
| NM_203405 | KRTAP26-1 | Keratin associated protein 26-1 | 0.041 |
| AK093561 | LOC100131792 | Hypothetical LOC100131792 | 0.035 |
| AK056534 | LOC145757 | Hypothetical LOC145757 | 0.016 |
| U72518 | LOC171220 | Destrin-2 pseudogene | 0.013 |
| BC047037 | LOC644714 | Hypothetical protein LOC644714 | 0.004 |
| XM_001129774 | LOC650157 | Similar to TRIM5/cypa fusion protein | 0.017 |
| XR_042505 | LOC652811 | Similar to adlican | 0.018 |
| NR_028093 | LPAL2 | Lipoprotein, Lp(a)-like 2 | 0.005 |
| NM_003954 | MAP3K14 | Mitogen-activated protein kinase kinase kinase 14 | 0.008 |
| NM_014048 | MKL2 | MKL/myocardin-like 2 | 0.049 |
| NM_005664 | MKRN3 | Makorin ring finger protein 3 | 0.045 |
| NM_002424 | MMP8 | Matrix metallopeptidase 8 (neutrophil collagenase) | 0.039 |
| NM_020191 | MRPS22 | Mitochondrial ribosomal protein S22 | 0.041 |
| NM_033054 | MYO1G | Myosin IG | 0.042 |
| EU000855 | NBLA00301 | Nbla00301 | 0.023 |
| NM_000908 | NPR3 | Natriuretic peptide receptor C/guanylate cyclase C (atrionatriuretic peptide receptor C) | 0.050 |
| NM_152745 | NXPH1 | Neurexophilin 1 | 0.031 |
| NM_013939 | OR10H2 | Olfactory receptor, family 10, subfamily H, member 2 | 0.029 |
| NM_001004478 | OR10Z1 | Olfactory receptor, family 10, subfamily Z, member 1 | 0.040 |
| NM_001004459 | OR1S2 | Olfactory receptor, family 1, subfamily S, member 2 | 0.013 |
| NM_001004693 | OR2T10 | Olfactory receptor, family 2, subfamily T, member 10 | 0.025 |
| NM_001001955 | OR4C13 | Olfactory receptor, family 4, subfamily C, member 13 | 0.036 |
| NR_002140 | OR6W1P | Olfactory receptor, family 6, subfamily W, member 1 pseudogene | 0.050 |
| NM_207339 | PAGE2 | P antigen family, member 2 (prostate associated) | 0.018 |
| NM_001142763 | PCDH15 | Protocadherin 15 | 0.012 |
| NM_019120 | PCDHB8 | Protocadherin beta 8 | 0.012 |
| NM_002630 | PGC | Progastricsin (pepsinogen C) | 0.024 |
| NM_003625 | PPFIA2 | Protein tyrosine phosphatase, receptor type, f polypeptide (PTPRF), interacting protein (liprin), alpha 2 | 0.023 |
| NM_001145014 | RFPL4A | Similar to Ret finger protein-like 4A; ret finger protein-like 4A | 0.041 |
| NM_000539 | RHO | Rhodopsin | 0.046 |
| NM_173642 | RIMKLA | Ribosomal modification protein rimk-like family member A | 0.021 |
| NR_002757 | RNU5B-1 | RNA, U5E small nuclear; RNA, U5A small nuclear; RNA, U5F small nuclear; RNA, U5B small nuclear 1; RNA, U5D small nuclear | 0.034 |
| NR_003572 | RPL23AP53 | Ribosomal protein L23a pseudogene 53 | 0.047 |
| NR_003011 | SCARNA15 | Small Cajal body-specific RNA 15 | 0.016 |
| NM_080475 | SERPINB11 | Serpin peptidase inhibitor, clade B (ovalbumin), member 11 (gene/pseudogene) | 0.045 |
| NM_207386 | SHISA6 | FLJ45455 protein | 0.007 |
| NM_152351 | SLC5A10 | Solute carrier family 5 (sodium/glucose cotransporter), member 10 | 0.033 |
| NM_145913 | SLC5A8 | Solute carrier family 5 (iodide transporter), member 8 | 0.009 |
| NR_004396 | SNORD1B | Small nucleolar RNA, C/D box 1A; small nucleolar RNA, C/D box 1B | 0.032 |
| NR_002602 | SNORD37 | Small nucleolar RNA, C/D box 37 | 0.050 |
| NM_001056 | SULT1C2 | Sulfotransferase family, cytosolic, 1C, member 2 | 0.034 |
| NM_145653 | TCEB3C | Transcription elongation factor B polypeptide 3C-like; transcription elongation factor B polypeptide 3C-like 2; transcription elongation factor B polypeptide 3C (elongin A3) | 0.041 |
| NM_001100817 | TCEB3CL | Transcription elongation factor B polypeptide 3C-like; transcription elongation factor B polypeptide 3C-like 2; transcription elongation factor B polypeptide 3C (elongin A3) | 0.036 |
| NM_182578 | THEM5 | Thioesterase superfamily member 5 | 0.006 |
| NM_138461 | TM4SF19 | Transmembrane 4 L six family member 19 | 0.049 |
| NM_178520 | TMEM105 | Transmembrane protein 105 | 0.028 |
| NM_003492 | TMEM187 | Transmembrane protein 187 | 0.045 |
| NM_016212 | TP53TG3 | Similar to TP53 target 3; TP53 target 3; similar to TP53TG3 protein | 0.006 |
| NM_006088 | TUBB2C | Tubulin, beta 2C | 0.047 |
| NM_006398 | UBD | Ubiquitin D | 0.033 |
| NM_007000 | UPK1A | Uroplakin 1A | 0.045 |
| NM_013378 | VPREB3 | Pre-B lymphocyte 3 | 0.023 |
| ENST00000338912 | VSIG7 | V-set and immunoglobulin domain containing 7; immunoglobulin heavy variable 1/OR15-5 pseudogene; immunoglobulin heavy variable 1/OR15-9 (non-functional) | 0.037 |
| NM_001037735 | ZNF630 | Zinc finger protein 630 | 0.015 |
| AK296954 | ZNF852 | Zinc finger protein 852; zinc finger protein 167 | 0.006 |
