# Supplementary material for: Identification of Target Genes Involved in Wound Healing Angiogenesis of Endothelial Cells with the Treatment of a Chinese 2-Herb Formula
Source: PLoS One. 2015 Oct 2;10(10):e0139342. doi: 10.1371/journal.pone.0139342 (PMC4591983; doi:10.1371/journal.pone.0139342)
Supplement: S2 Table — (DOCX) [file pone.0139342.s002.docx]

**S2 table. Differentially expressed genes in NF3-treated HUVEC versus control at 16 h from microarray analysis.**

| Accession Number | Gene Symbol | Gene Name | P Value |
| --- | --- | --- | --- |
| NM_022437 | ABCG8 | ATP-binding cassette, sub-family G (WHITE), member 8 | 0.002 |
| NM_001012659 | ARGFX | Arginine-fifty homeobox | 0.012 |
| NM_001042519 | C2orf88 | Chromosome 2 open reading frame 88 | 0.035 |
| AJ420538 | C6orf130 | Chromosome 6 open reading frame 130 | 0.002 |
| NM_033124 | CCDC65 | Coiled-coil domain containing 65 | 0.022 |
| NM_019083 | CCDC76 | Coiled-coil domain containing 76 | 0.037 |
| NM_006584 | CCT6B | Chaperonin containing TCP1, subunit 6B (zeta 2) | 0.041 |
| NR_003595 | CDC14C | CDC14 cell division cycle 14 homolog C (S. Cerevisiae) | 0.022 |
| NM_021153 | CDH19 | Cadherin 19, type 2 | 0.028 |
| NM_004076 | CRYBB3 | Crystallin, beta B3 | 0.027 |
| NM_001082 | CYP4F2 | Cytochrome P450, family 4, subfamily F, polypeptide 2 | 0.030 |
| NM_178470 | DCAF12L1 | WD repeat domain 40B | 0.002 |
| NR_003668 | DEFB109P1B | Defensin, beta 109, pseudogene 1; defensin, beta 109, pseudogene 1B | 0.048 |
| NM_001037500 | DEFB124 | Defensin, beta 124 | 0.015 |
| NM_198449 | EMB | Embigin homolog (mouse) | 0.022 |
| NM_001166005 | EPB41 | Erythrocyte membrane protein band 4.1 (elliptocytosis 1, RH-linked) | 0.041 |
| NM_001029888 | FAM24A | Family with sequence similarity 24, member A | 0.031 |
| AK131225 | FLJ16126 | Hypothetical LOC645010 | 0.000 |
| AK092009 | FLJ34690 | Hypothetical protein FLJ34690 | 0.041 |
| NR_015358 | FLJ43390 | Hypothetical LOC646113 | 0.027 |
| NM_017412 | FZD3 | Frizzled homolog 3 (Drosophila) | 0.024 |
| NM_001496 | GFRA3 | GDNF family receptor alpha 3 | 0.017 |
| NM_007327 | GRIN1 | Glutamate receptor, ionotropic, N-methyl D-aspartate 1 | 0.029 |
| NM_178171 | GSDMA | Gasdermin A | 0.037 |
| NR_003081 | GSTTP1 | Similar to Glutathione S-transferase theta-4; glutathione S-transferase theta pseudogene 1 | 0.030 |
| NM_002099 | GYPA | Glycophorin A (MNS blood group) | 0.009 |
| NR_026978 | HAUS1 | HAUS augmin-like complex, subunit 1 | 0.029 |
| NM_138737 | HEPH | Hephaestin | 0.027 |
| NM_017416 | IL1RAPL2 | Interleukin 1 receptor accessory protein-like 2 | 0.023 |
| NM_002297 | LCN1 | Lipocalin 1-like 1; lipocalin 1 (tear prealbumin) | 0.009 |
| AK096606 | LOC100131170 | Hypothetical LOC100131170 | 0.023 |
| AK127783 | LOC400968 | Hypothetical LOC400968 | 0.042 |
| BX648692 | LOC401410 | Hypothetical LOC401410 | 0.036 |
| AK096082 | LOC646976 | Hypothetical LOC646976 | 0.026 |
| NR_003671 | LOC728024 | Hcg1640171 | 0.044 |
| NM_020737 | LRFN2 | Leucine rich repeat and fibronectin type III domain containing 2 | 0.010 |
| NM_015613 | LRIT1 | Leucine-rich repeat, immunoglobulin-like and transmembrane domains 1 | 0.036 |
| NM_006533 | MIA | Melanoma inhibitory activity | 0.014 |
| NR_029674 | MIR132 | Microrna 132 | 0.013 |
| NR_029502 | MIR28 | Microrna 28 | 0.047 |
| NR_029482 | MIRLET7E | Microrna let-7e | 0.003 |
| NM_022045 | MTBP | Mdm2, transformed 3T3 cell double minute 2, p53 binding protein (mouse) binding protein, 104kda | 0.037 |
| NM_001040105 | MUC17 | Mucin 17, cell surface associated | 0.048 |
| NR_015370 | NCRNA00219 | Chromosome 5 open reading frame 26 | 0.007 |
| NM_145007 | NLRP11 | NLR family, pyrin domain containing 11 | 0.011 |
| NM_005123 | NR1H4 | Nuclear receptor subfamily 1, group H, member 4 | 0.003 |
| NM_001004462 | OR10G4 | Olfactory receptor, family 10, subfamily G, member 4 | 0.039 |
| NM_001004297 | OR13A1 | Olfactory receptor, family 13, subfamily A, member 1 | 0.006 |
| NM_001004485 | OR13F1 | Olfactory receptor, family 13, subfamily F, member 1 | 0.026 |
| NM_007160 | OR2H2 | Olfactory receptor, family 2, subfamily H, member 2 | 0.046 |
| NM_001004687 | OR2L3 | Olfactory receptor, family 2, subfamily L, member 3 | 0.034 |
| NR_028067 | OR4N3P | Olfactory receptor, family 4, subfamily N, member 3 pseudogene | 0.026 |
| NM_172194 | OR4Q3 | Olfactory receptor, family 4, subfamily Q, member 3 | 0.046 |
| NM_012375 | OR52A1 | Olfactory receptor, family 52, subfamily A, member 1 | 0.016 |
| NM_001005497 | OR6C75 | Olfactory receptor, family 6, subfamily C, member 75 | 0.011 |
| NM_001005202 | OR8K3 | Olfactory receptor, family 8, subfamily K, member 3 | 0.017 |
| NM_001167902 | PGPEP1L | Pyroglutamyl-peptidase 1-like | 0.028 |
| NM_001101387 | PIRT | Phosphoinositide-interacting regulator of transient receptor potential channels | 0.037 |
| NM_020227 | PRDM9 | PR domain containing 9 | 0.003 |
| NM_000948 | PRL | Prolactin | 0.021 |
| NM_171998 | RAB39B | RAB39B, member RAS oncogene family | 0.036 |
| NR_002939 | RUNDC2C | RUN domain containing 2B; RUN domain containing 2C | 0.039 |
| NM_181885 | RXFP4 | Relaxin/insulin-like family peptide receptor 4 | 0.028 |
| NM_005980 | S100P | S100 calcium binding protein P | 0.021 |
| NM_003469 | SCG2 | Secretogranin II (chromogranin C) | 0.033 |
| NM_052931 | SLAMF6 | SLAM family member 6 | 0.030 |
| NM_001135181 | SLC5A9 | Solute carrier family 5 (sodium/glucose cotransporter), member 9 | 0.001 |
| NR_002993 | SNORA35 | Small nucleolar RNA, H/ACA box 35 | 0.023 |
| NR_003708 | SNORA70C | Small nucleolar RNA, H/ACA box 70C (retrotransposed); small nucleolar RNA, H/ACA box 70; small nucleolar RNA, H/ACA box 70B (retrotransposed) | 0.045 |
| AB096175 | SP5 | Sp5 transcription factor | 0.020 |
| NM_014767 | SPOCK2 | Sparc/osteonectin, cwcv and kazal-like domains proteoglycan (testican) 2 | 0.020 |
| NM_001105578 | SYCE2 | Synaptonemal complex central element protein 2 | 0.003 |
| NR_028511 | TAAR3 | Trace amine associated receptor 3 (gene/pseudogene) | 0.023 |
| NM_176881 | TAS2R39 | Taste receptor, type 2, member 39 | 0.019 |
| NM_058222 | TECTB | Tectorin beta | 0.026 |
| NM_003220 | TFAP2A | Transcription factor AP-2 alpha (activating enhancer binding protein 2 alpha) | 0.005 |
| NM_021992 | TMSB15A | Thymosin beta 15a; thymosin beta 15B | 0.030 |
| NM_001011658 | TRAPPC2 | Trafficking protein particle complex 2; trafficking protein particle complex 2 pseudogene 1 | 0.002 |
| NM_016388 | TRAT1 | T cell receptor associated transmembrane adaptor 1 | 0.026 |
| NM_004616 | TSPAN8 | Tetraspanin 8 | 0.028 |
| NM_152574 | TTC39B | Tetratricopeptide repeat domain 39B | 0.024 |
| NM_005783 | TXNDC9 | Thioredoxin domain containing 9 | 0.045 |
| NM_003372 | VBP1 | Von Hippel-Lindau binding protein 1 | 0.018 |
| NM_173858 | VN1R5 | Vomeronasal 1 receptor 5 | 0.028 |
| NM_016516 | VPS54 | Vacuolar protein sorting 54 homolog (S. Cerevisiae) | 0.026 |
| NM_016303 | WBP5 | WW domain binding protein 5 | 0.018 |
| NM_080753 | WFDC10A | WAP four-disulfide core domain 10A | 0.021 |
| NM_001099270 | ZBTB34 | Zinc finger and BTB domain containing 34 | 0.002 |
| NM_016220 | ZNF107 | Zinc finger protein 107 | 0.024 |
| NM_003441 | ZNF141 | Zinc finger protein 141 | 0.014 |
| NM_033468 | ZNF257 | Zinc finger protein 257 | 0.009 |
| NM_020951 | ZNF529 | Zinc finger protein 529 | 0.006 |
| NM_033196 | ZNF682 | Zinc finger protein 682 | 0.035 |
| NM_001004328 | ZNF705A | Zinc finger protein 705A | 0.030 |
| NM_021998 | ZNF711 | Zinc finger protein 711 | 0.030 |
| NM_001144989 | ZNF814 | Zinc finger protein 814 | 0.018 |
